# Supplementary material for: Leveraging Machine Learning-Guided Molecular Simulations Coupled with Experimental Data to Decipher Membrane Binding Mechanisms of Aminosterols
Source: J Chem Theory Comput. 2024 Jul 9;20(18):8279–89. doi: 10.1021/acs.jctc.4c00127 (PMC11447954; doi:10.1021/acs.jctc.4c00127)
Supplement: Supplementary file 1 — ct4c00127_si_001.pdf [file ct4c00127_si_001.pdf]

Supporting Information

Leveraging Machine Learning-Guided Molecular Simulations Coupled with Experimental Data to Decipher Membrane Binding Mechanisms of Aminosterols

Authors: Stefano Muscat[1], Silvia Errico[2], Andrea Danani[1], Fabrizio Chiti[2], Gianvito Grasso[1,*]

[1]Dalle Molle Institute for Artificial Intelligence IDSIA USI-SUPSI, Via la Santa 1, 6962 Lugano-Viganello, Switzerland;

[2] Department of Experimental and Clinical Biomedical Sciences, Section of Biochemistry, University of Florence, 50134 Florence, Italy

*Corresponding Author: gianvito.grasso@supsi.ch

S1 - Trodusquemine and squalamine bond terms optimization.

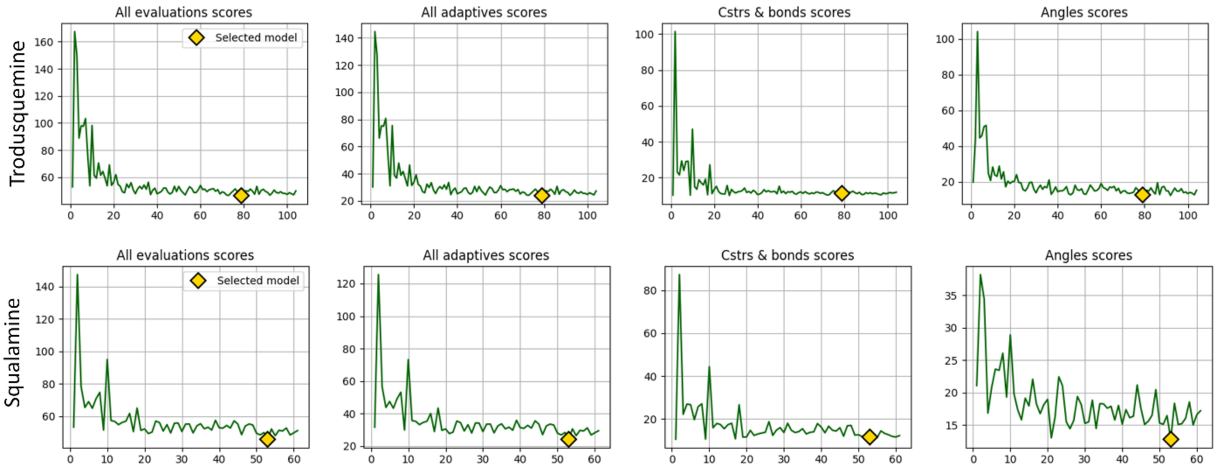

Figure S1: Total optimization scores over iterations.

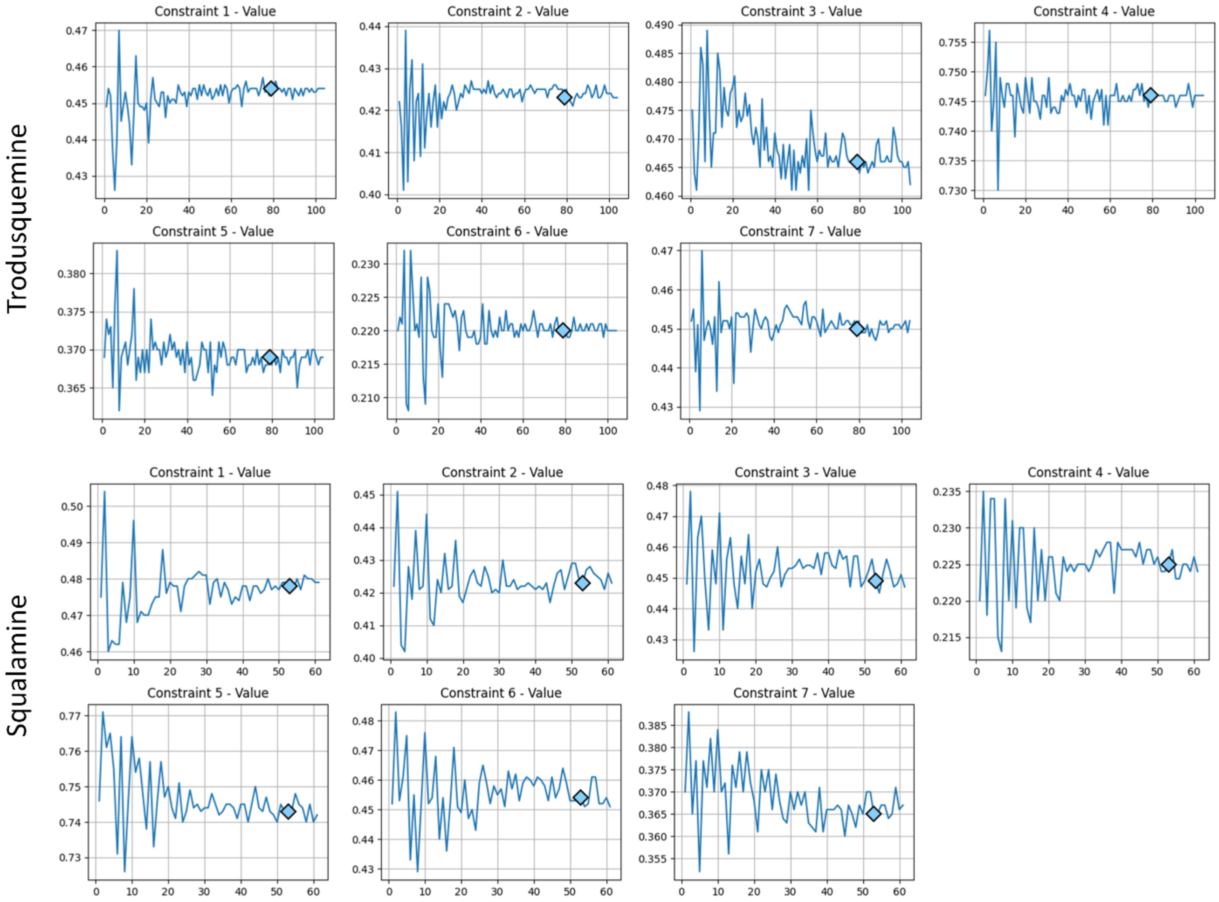

Figure S2: Constraints optimization over iterations.

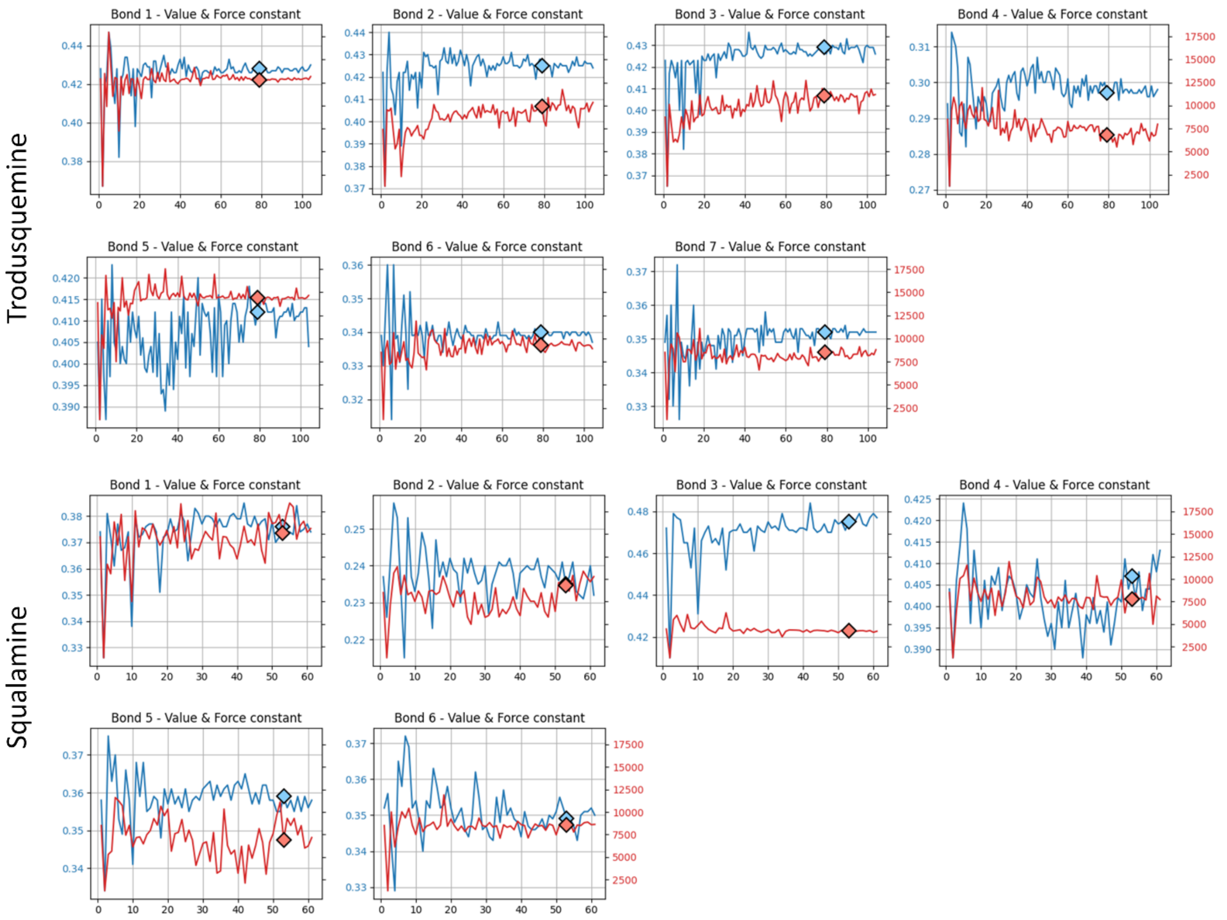

Figure S3: Bonds optimization over iterations.

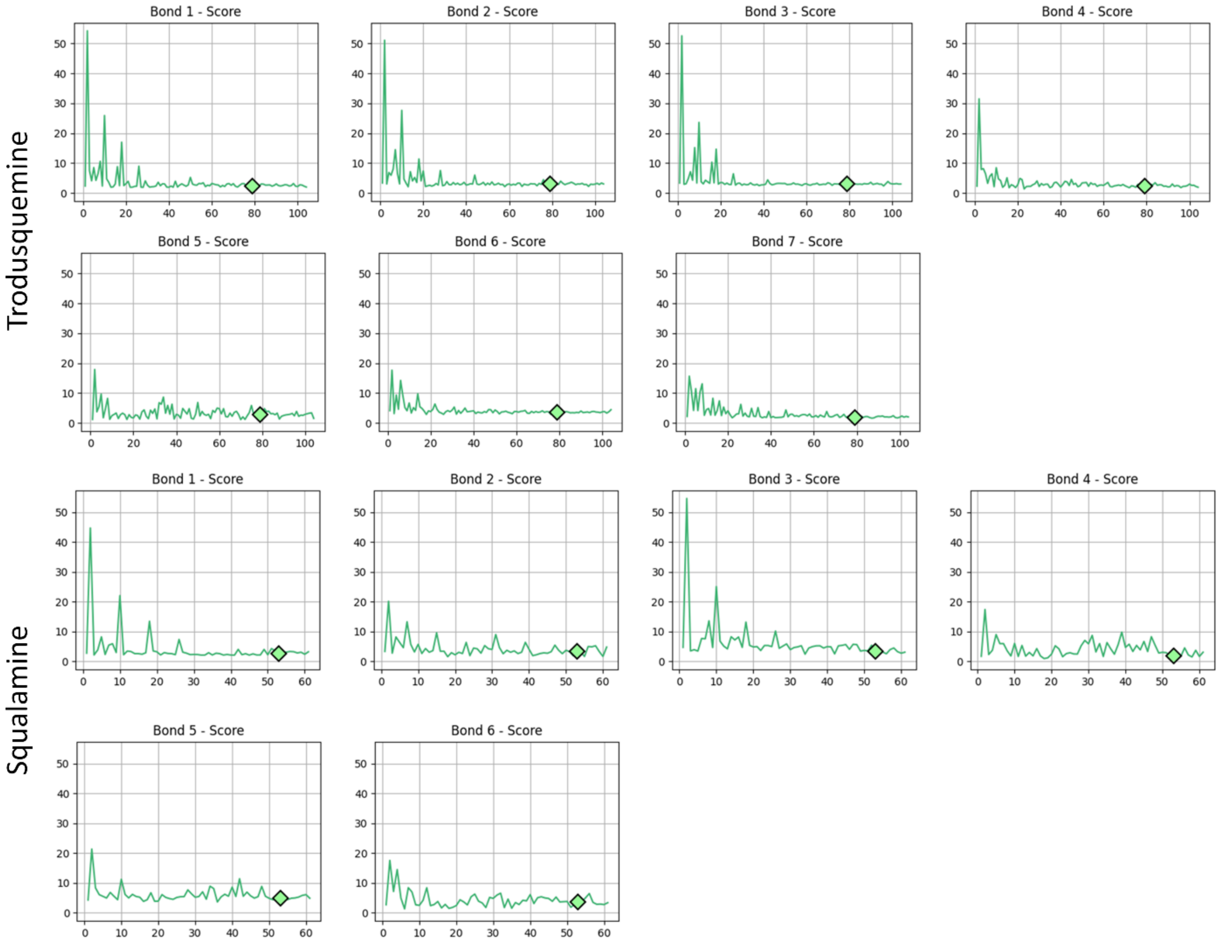

Figure S4:Summary of the bond scores over iterations.

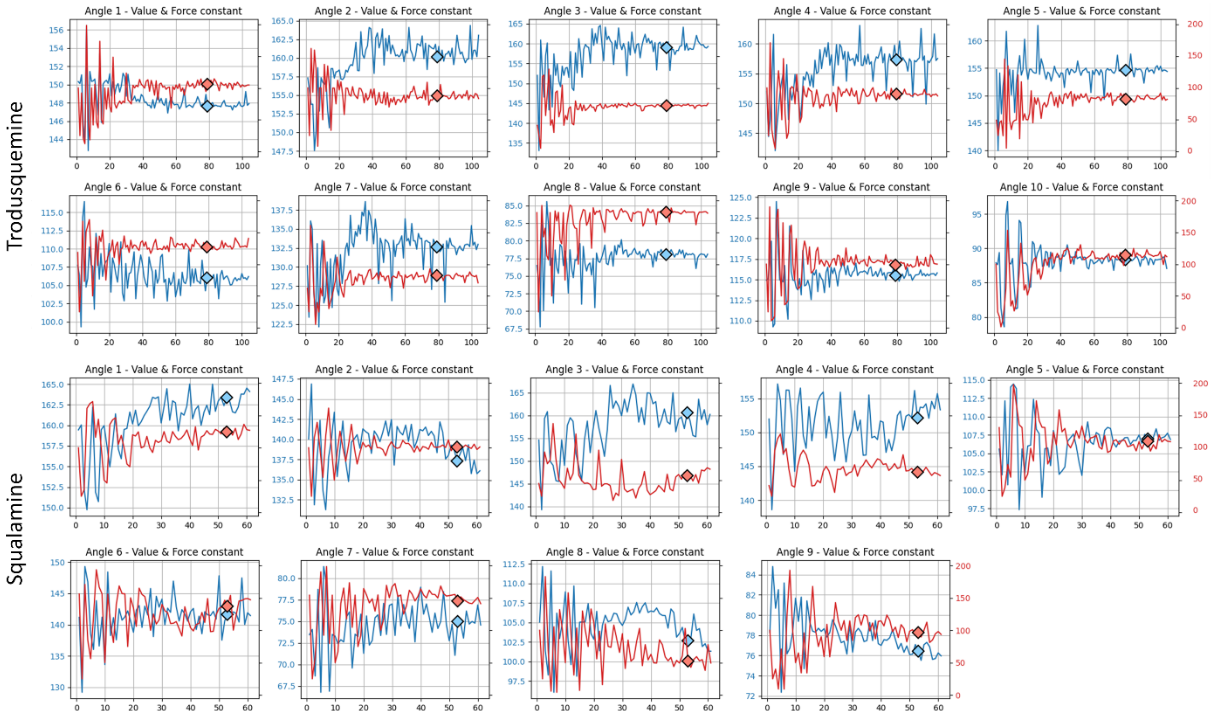

Figure S5:Angles optimization over iterations.

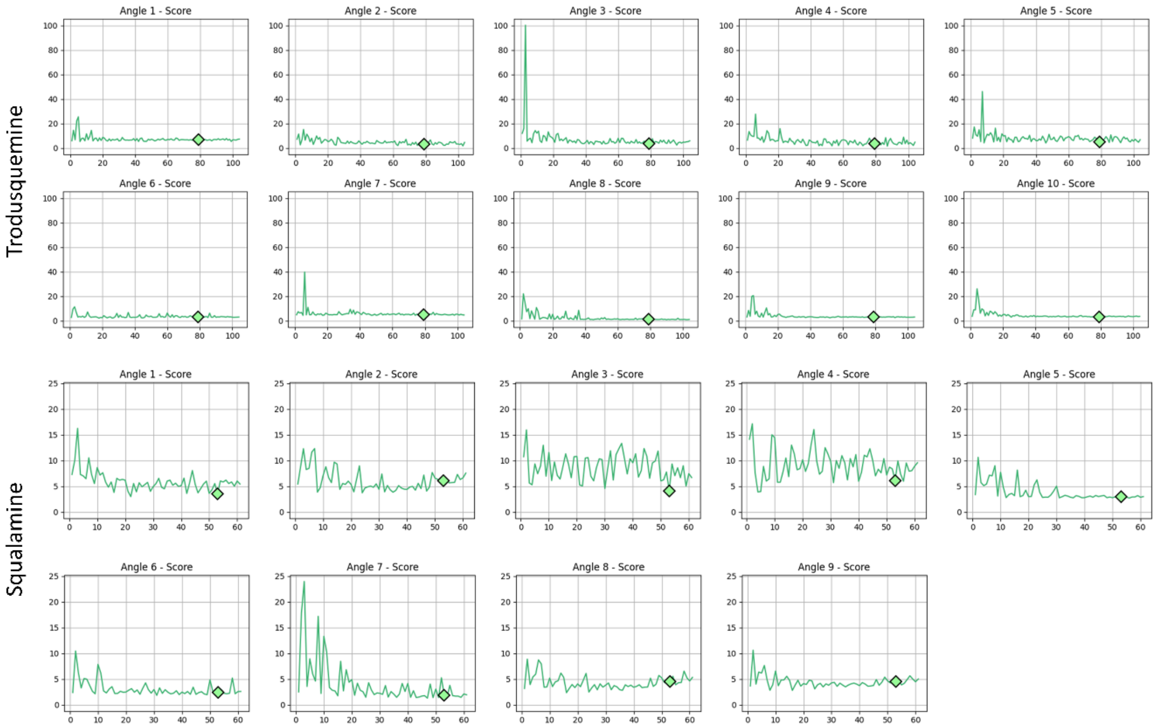

Figure S6: Summary of angles over iterations.

S2 – OPES multithermal convergence

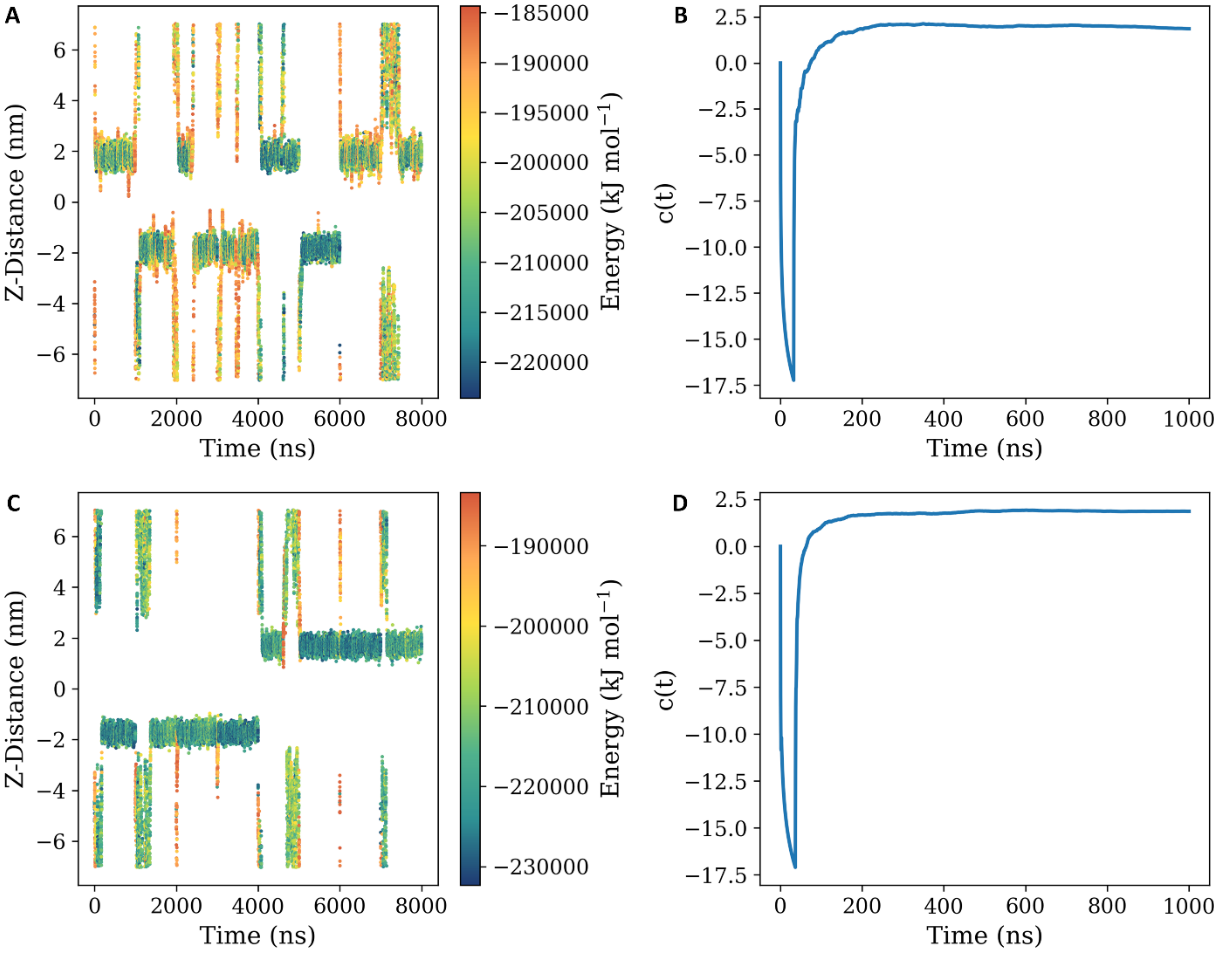

Figure S7: Time evolution of the normal distance between the center of mass of aminosterol (trodusquemine in panel A and squalamine in panel C) and the center of the cell membrane, colored by the potential energy of the molecular system. Time evolution of the OPES multithermal bias shared between 8 replicas that assesses the convergence of the simulation of trodusquemine (panel B) or squalamine (panel D) system.

S2 – Deep-TICA training and validation

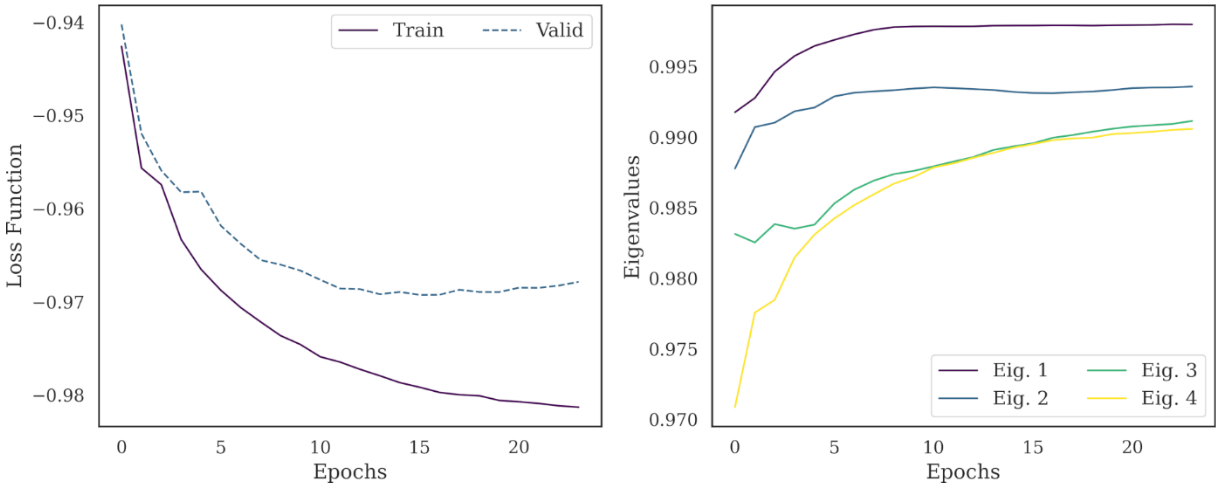

Figure S8: Loss function for TICA training (left panel) and eigenvalue of first TICA vectors (right panel) vs epochs of trodusquemine system.

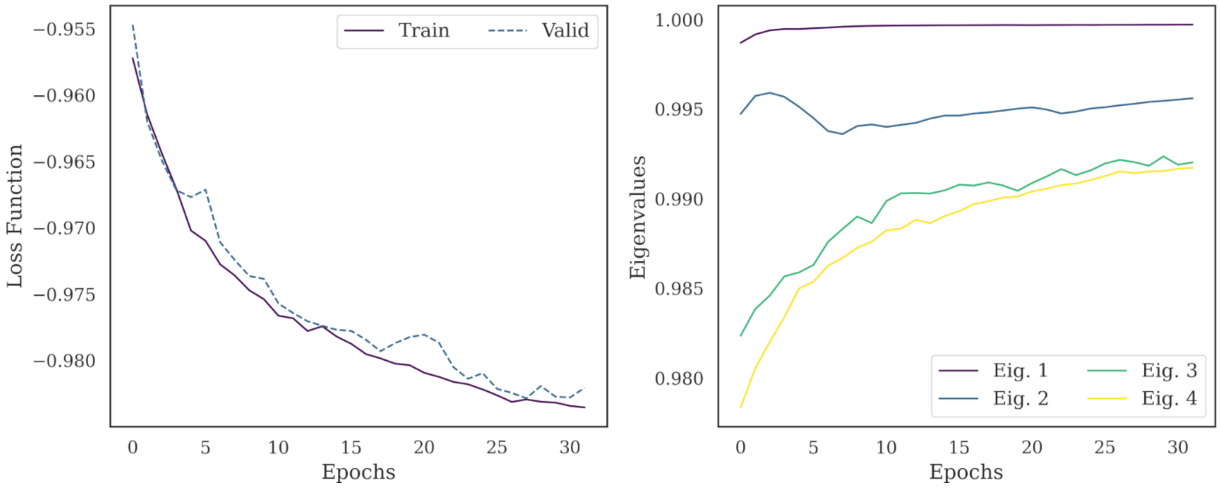

Figure S9: Loss function for TICA training (left panel) and eigenvalue of first TICA vectors (right panel) vs epochs of squalamine system.

S3 – FES 2D

A

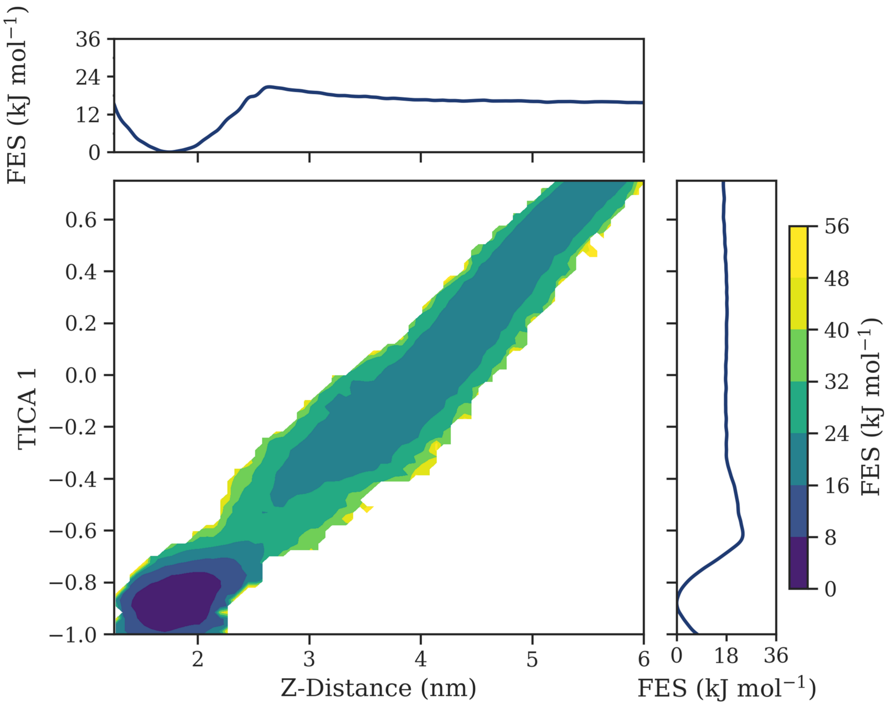

B

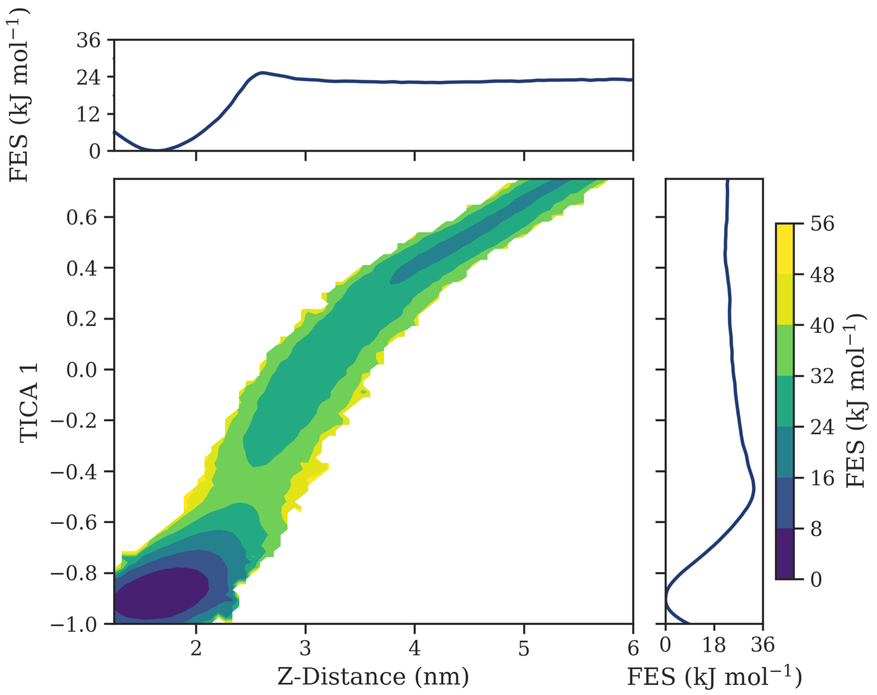

Figure S10: The FES of TRO (panel A) and SQ (panel B) is illustrated as a function of the z-distance from the membrane center and the Deep-TICA 1 CV. The FES projections along the corresponding axis are displayed in the upper and right panels.
